# Facile One-Step Heat Treatment of Cu Foil for Stable Anode-Free Li Metal Batteries

**DOI:** 10.3390/molecules28020548

**Published:** 2023-01-05

**Authors:** Jie Chen, Linna Dai, Pei Hu, Zhen Li

**Affiliations:** 1 School of Science, Hubei University of Technology, Wuhan 430068, China; 2 State Key Laboratory of Material Processing and Die and Mold Technology, School of Materials Science and Engineering, Huazhong University of Science and Technology, Wuhan 430074, China

**Keywords:** anode-free Li metal batteries, current collector modification, anode interface, solid electrolyte interphase

## Abstract

The anode-free lithium metal battery (AFLMB) is attractive for its ultimate high energy density. However, the poor cycling lifespan caused by the unstable anode interphase and the continuous Li consumption severely limits its practical application. Here, facile one-step heat treatment of the Cu foil current collectors before the cell assembly is proposed to improve the anode interphase during the cycling. After heat treatment of the Cu foil, homogeneous Li deposition is achieved during cycling because of the smoother surface morphology and enhanced lithiophilicity of the heat-treated Cu foil. In addition, Li_2_O-riched SEI is obtained after the Li deposition due to the generated Cu_2_O on the heat-treated Cu foil. The stable anode SEI can be successfully established and the Li consumption can be slowed down. Therefore, the cycling stability of the heat-treated Cu foil electrode is greatly improved in the Li|Cu half-cell and the symmetric cell. Moreover, the corresponding LFP|Cu anode-free full cell shows a much-improved capacity retention of 62% after 100 cycles, compared to that of 43% in the cell with the commercial Cu foil. This kind of facile but effective modification of current collectors can be directly applied in the anode-free batteries, which are assembled without Li pre-deposition on the anode.

## 1. Introduction

Lithium metal batteries (LMBs) have received board interest mainly due to the highest theoretical specific capacity (3860 mAh g^−1^) and the lowest redox potential (−3.04 V vs. the standard hydrogen electrode) of Li metal anodes [1,2,3,4]. AFLMB, whose Li source is provided only by the cathode, can be the ultimate choice among all the lithium metal batteries due to its high energy density and easy fabrication [5,6,7]. When the cathode is determined, the anode-free system achieves the highest gravimetric and volumetric densities among the combination options that use graphite, Sn, Si or Li metal anodes [8]. Avoiding the direct use of Li metal simplifies the battery fabrication and improves the security.

However, the AFLMB suffers from the predicaments caused by the extremely high reactivity of Li metal and colossal volume change during the repeated Li cycling, including the growth of Li dendrites, the generation and accumulation of dead Li, and the side reactions between metallic Li and the electrolyte [9,10,11]. Stable anode SEI is hard to establish and the active lithium is consumed continuously, which accelerates the battery failure [12]. The AFLMB without modification usually falls below 50% capacity retention in fewer than 50 cycles, which is far from the commercial application [5].

To improve the performance of AFLMB, many strategies have been proposed in recent years, such as the modification of current collectors [13,14,15,16], the electrolyte optimization [17,18,19,20], the lithium compensation in the cathode [21,22,23], and the optimization of external conditions (the mechanical pressure [24,25], the temperature [26,27], the cycling conditions [28], and so on). In AFLMB, it is worth noting that the initial lithium deposition morphology and formed solid electrolyte interphase (SEI) formed on the current collectors can influence the long-term evolution of the Li metal anode [29,30,31,32], which determines the cycle life of the batteries to a large extent. Thus, the properties of current collectors can play a crucial part in anode stabilization design. The “charge-induced growth model” reveals that the charge accumulation is beneficial to early Li dendrite growth [33]. Considering the “tip effect” that a protrusion on the electrode exhibits a stronger electrical field and gathers more charges [34,35], the surface structure of the current collectors should be smooth and flat. Moreover, whether the current collectors are lithiophilic or lithiophobic influences the Li nucleation pattern, which has an important effect on the lithium deposition morphology [29], and the current collectors should better be lithiophilic. The surface composition of some modified current collectors can also regulate the SEI through the reaction with the deposited Li, such as the content of LiF or Li_2_O in SEI, which can be improved by modifying the current collectors with active fluoric or oxygenic species, while LiF and Li_2_O are widely known to regulate the Li^+^ transfer and enhance the structural modulus [36,37,38,39]. The rational design of the reaction can contribute to building high-quality SEI.

In this work, a facile one-step heat treatment method is proposed to modify the surface structure, lithiophilicity, and even surface composition of the commercial Cu current collectors, and the optimal heat treatment time is identified to be 10 min. The heat-treated Cu foil shows a flatter surface morphology with fewer fluctuations than the commercial Cu foil, which avoids the charge accumulation and controls the lithium-ion flux. Cu_2_O is found to generate on the surface and the lithiophilicity is enhanced after the heat treatment. The Cu_2_O is demonstrated to transform into Li_2_O after Li deposition, constructing Li_2_O-riched SEI and improving the anode stability. As a result, when the 3.5 mAh cm^−2^ areal capacity LiFePO_4_ (LFP) cathode is used, the anode-free full cell with the heat-treated Cu foil maintains high capacity retentions of 62% and 47% after 100 and 200 cycles, compared to that of 43% and 31% in the cell with the commercial Cu foil.

## 2. Results and Discussion

**Figure 1:**
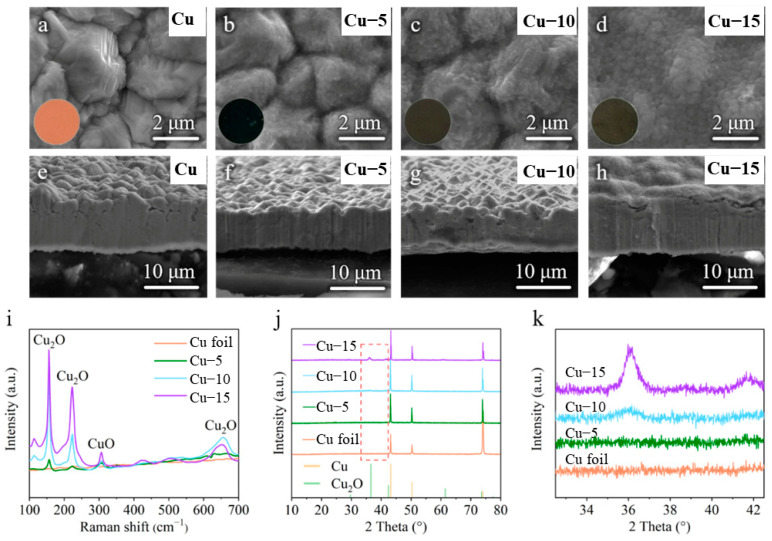
(**a**–**d**) Surface and (**e**–**h**) cross-sectional SEM images of the (**a**,**e**) commercial Cu foil, (**b**,**f**) Cu–5, (**c**,**g**) Cu–10 and (**d**,**h**) Cu–15. The inset is the corresponding optical photos. (**i**) Raman spectra of different Cu foils. (**j**) XRD patterns and (**k**) the partially enlarged drawing at 33–42° of different Cu foils.

The commercial Cu foils were heat-treated by a facile one-step method. They were heated at 320 ℃ in the atmosphere for 5, 10 and 15 min for comparison. The color of the Cu foils was changed from purplish red (Cu foil) to dark blue (Cu–5) and dark brown (Cu–10 and Cu–15) after the heat treatment, as shown in the insets of Figure 1a–d. The SEM image of the Cu foil shows rough morphology with distinct grains (Figure 1a), while the grains become blurred and the surface morphology becomes smooth gradually with the extension of heat treatment time (Figure 1b–d). The cross-sectional SEM images of the Cu foils in Figure 1e–h also confirm the trend of the morphology change.

To identify the changes in surface composition of the Cu foils, the Raman spectra and the XRD tests were performed. Raman spectrum is a kind of molecule scattering spectroscopy, which is characterized by the frequency excursion caused by the interactions of the molecule and photon to show the information of molecule. As shown in Figure 1i, the commercial Cu foil shows no signal because there is no molecular structure in the metal elementary substance, while the heat-treated Cu foils reveal obvious characteristic peaks of Cu_2_O at 156, 222, 654 cm^−1^ and a weak characteristic peak of CuO at 305 cm^−1^ [40,41]. The XRD patterns of different samples in Figure 1j both show three strong characteristic peaks of Cu at 43°, 51° and 74° (PDF#04-0836), which means the main ingredient of the current collectors is still Cu. However, the intensity of the characteristic peaks of Cu_2_O at 36° and 42° (PDF#05-0667) increases gradually, as shown in the partially enlarged drawing of Figure 1k, and no characteristic peak of CuO is found. Taking into account the results of SEM images, the Raman spectra and the XRD patterns, it is believed that a thin layer of Cu_2_O with little CuO is generated on the surface of the Cu foils after the heat treatment and the amount of Cu_2_O increases with the extension of the heat treatment time.

**Figure 2:**
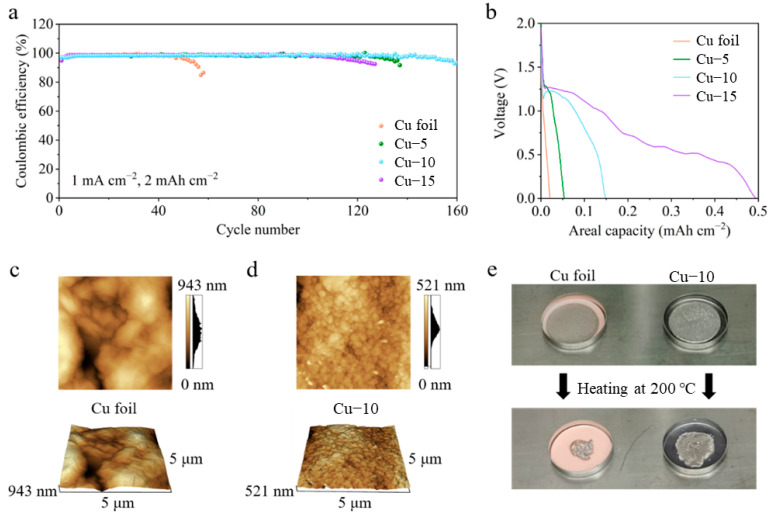
(**a**) Coulombic efficiency and (**b**) the first discharge profiles of the cells using the commercial Cu foil, Cu–5, Cu–10 or Cu–15. AFM images of the (**c**) commercial Cu foil and (**d**) Cu–10. (**e**) Optical photos of Li foil on the commercial Cu foil or Cu–10 before and after heating at 200 °C.

CE tests of different Cu foils in Li|Cu half-cells were performed to reveal the optimum heat treatment time in Figure 2a. With the current density of 1 mA cm^−2^ and the areal capacity of 2 mAh cm^−2^, the half-cell with the Cu–10 shows the best cycle life of 160 cycles. A total of 137 and 127 cycles are achieved in the cells with the Cu–5 and Cu–15, while only 56 cycles are achieved when the commercial Cu foil is used. It is worth noting that there is a certain amount of discharge capacity above 0 V in the first cycle, as shown in Figure 2b, which may mean the reaction between Cu_2_O and the deposited Li. However, the reaction is almost irreversible in the following charge process and causes the loss of the active Li, which is a severe issue in the lithium-limited AFLMB. Thus, the discharge capacity above 0 V should be as little as possible. The amount of Cu_2_O is critical. The effect is limited with little Cu_2_O, and active Li is lost severely with much Cu_2_O. Overall speaking, Cu–10 samples are better than Cu–5 and Cu–15. The optimum heat treatment is chosen to be 10 min through the above analysis. Only the characterization and electrochemical tests of the commercial Cu foil and Cu–10 are compared in the following part.

The atomic force microscope was applied to further characterize the change in the surface morphology of the Cu foils, as shown in Figure 2c,d. The AFM images of the commercial Cu foil show a longitudinal fluctuation of 943 nm in a 5 μm × 5 μm area, which is much larger than that of 521 nm in the Cu–10. In addition, the grains seem to be smaller after the heat treatment. Smaller longitudinal fluctuation and grains mean a smoother surface morphology which is beneficial to the homogenization of charges. Figure 2e shows the lithiophilicity tests of the Cu foil and the Cu–10. Two of the same Li foils were put on different Cu foils and heated at 200 °C simultaneously on the hot plate. The molten Li on the commercial Cu foil turns into a sphere so as to decrease the contact area with the lithiophobic surface, while the molten Li completely spreads on the Cu–10. The obvious contrast reveals the improvement of the lithiophilicity, which is beneficial to the Li nucleation on the current collectors.

**Figure 3:**
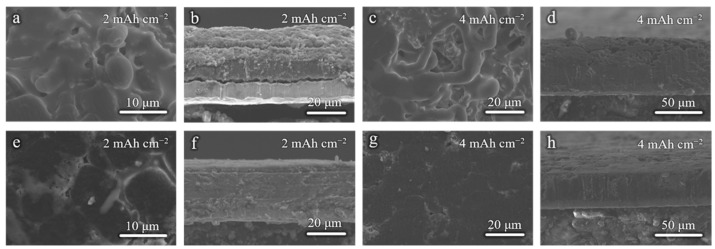
Surface and cross-sectional SEM images of (**a**,**b**,**e**,**f**) 2 mAh cm^−2^ and (**c**,**d**,**g**,**h**) 4 mAh cm^−2^ Li deposition on the (**a**–**d**) commercial Cu foil and (**e**–**h**) Cu–10.

To demonstrate the effect of modified Cu foils on the Li deposition morphology, the Li|Cu half-cells were cycled at 1 mA cm^−2^ for 5 cycles and disassembled for SEM tests. Figure 3a,b shows the surface and cross-section SEM images when the 2 mAh cm^−2^ areal capacity is applied. Due to the rough and lithiophobic surface of the commercial Cu foil, the Li deposition morphology is uneven with many protrusions (Figure 3a,b), which will further cause the growth of Li dendrites and the instability of the anode interphase. As shown in Figure 3c,d, when the areal capacity is increased to 4 mAh cm^−2^, the Li dendrites are evident on the surface. It can be speculated that there must have been many side reactions occurring at the anode. The Li deposition morphology is dense and homogeneous when the Cu–10 is used, whether the 2 mAh cm^−2^ or 4 mAh cm^−2^ areal capacity is applied (Figure 3e–h). This is benefited from the smoother surface morphology and the lithiophilicity of Cu–10, which is better for the homogenization and the nucleation of Li-ions.

**Figure 4:**
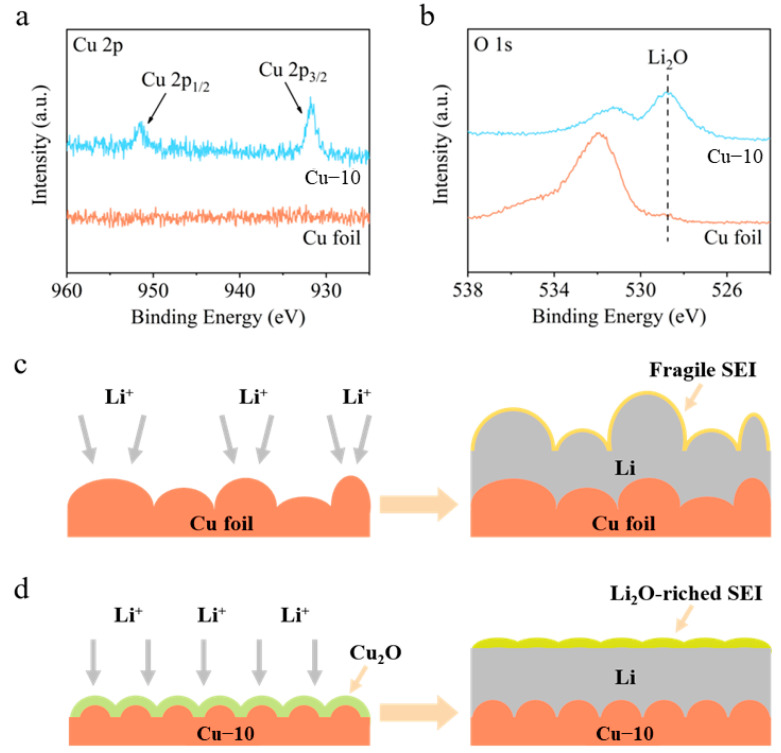
(**a**) Cu 2p and (**b**) O 1s XPS spectra of the surface of the commercial Cu foil and Cu–10 after Li deposition. Schematic illustrations of Li deposition on the (**c**) commercial Cu foil and (**d**) Cu–10.

XPS spectra were employed to analyze the components of SEI on the cycled Li-Cu anodes after five cycles. As shown in Figure 4a, the Cu 2p spectrum of the commercial Cu foil electrode shows no obvious peaks, demonstrating that there is no Cu in the SEI and the thickness of the deposited Li is greater than the depth of XPS detection (a few nanometers). However, the Cu 2p spectrum of the Cu–10 electrode shows two peaks at 931.8 and 951.5 eV, corresponding to Cu 2p_3/2_ and Cu 2p_1/2_, respectively. In addition, the O 1s spectra in Figure 4b show a strong peak at 528.8 eV of Li_2_O for the Cu–10 electrode, while showing a negligible peak for the commercial Cu foil electrode. In consideration of the Cu 2p and O 1s spectra, it is believed that after the Li deposition, the Cu_2_O on the Cu–10 transforms into Cu and Li_2_O, which further participate in the formation of SEI.

Based on the above analysis, a comprehensive modification mechanism of the current collector is proposed when the commercial Cu foil is heat-treated (Figure 4c,d). On the one hand, the heat treatment makes the surface morphology smoother before the Li deposition, which is beneficial to the homogenization of charges and Li-ions’ flux. Moreover, the Cu_2_O generated on the surface increases the lithiophilicity, improving the Li nucleation. On the other hand, the Cu_2_O can transform into Cu and Li_2_O after Li deposition, which promoted the formation of Li_2_O-riched SEI on the Cu–10 electrode rather than the fragile SEI on the commercial Cu foil electrode. Li_2_O is reported to be a favorable component of SEI to facilitate Li^+^ transportation [37], prevent excessive decomposition of the electrolyte [42], and provide the required mechanical strength [43]. Consequently, uniform Li deposition and stable anode interphase are gained on the Cu–10 electrode.

**Figure 5:**
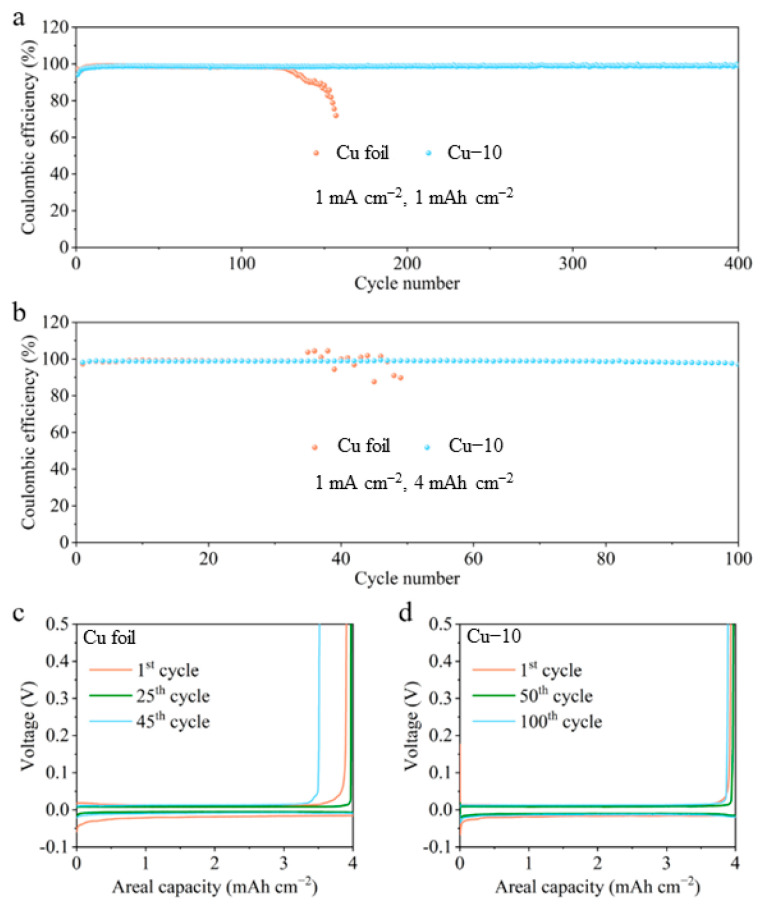
Coulombic efficiency of the cells using the commercial Cu foil and Cu–10 at 1 mA cm^−2^ with the capacity of (**a**) 1 mAh cm^−2^ and (**b**) 4 mAh cm^−2^. The corresponding voltage profiles of the cells using the (**c**) commercial Cu foil and (**d**) Cu–10 at different cycles with the capacity of 4 mAh cm^−2^.

The effectiveness of the modified current collector in improving the stability of the Li-Cu anode was further verified by the electrochemical tests. Figure 5 shows the CE tests of different Cu foil electrodes at a current density of 1 mA cm^−2^ with various areal capacities. In Figure 5a, the CE values of the commercial Cu foil electrode reach 98.9% at 1 mAh cm^−2^ in the initial cycles but then drop to 98.4%, and finally fail in 130 cycles. The CE values of the Cu–10 electrode stabilize at 98.7% in a few cycles and eventually maintain at 99.0% after 400 cycles. As shown in Figure 5b–d, when the areal capacity is increased to 4 mAh cm^−2^, the cell with the commercial Cu foil can only stabilize for 40 cycles and the plating/stripping overpotential gradually grow larger, while stable 100 cycles are achieved and almost constant overpotential is maintained in the cell with the Cu–10. The huge difference in the cycling stability is connected to the interphase between the deposited Li and the electrolyte. Poor interphase causes the growth of Li dendrites, the formation of the dead Li and the continuous side reaction, which accelerates the failure of the cell using the commercial Cu foil in the CE test. The smooth surface morphology and the lithiophilicity of the Cu–10, as well as the Li_2_O-riched SEI on the Cu–10 electrode, facilitate stable interphase, further guaranteeing the outstanding performance in the CE test.

**Figure 6:**
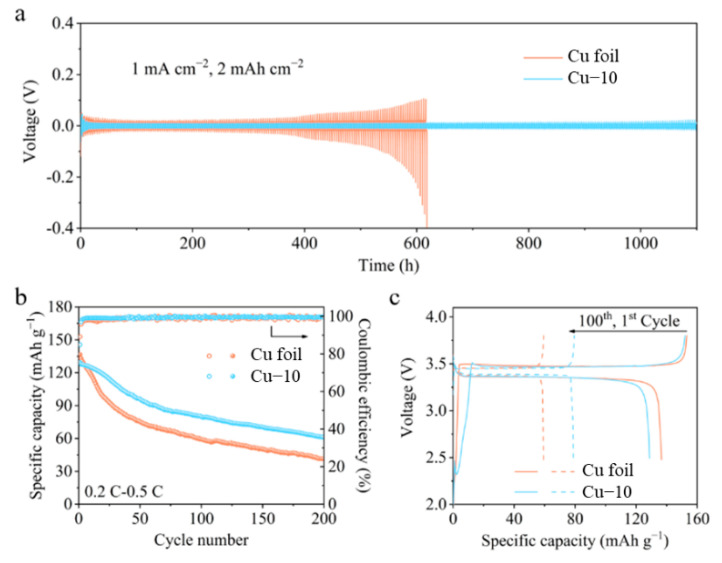
(**a**) Voltage profiles of the symmetric cells using the commercial Cu foil and Cu–10. (**b**) Cycling performance and (**c**) galvanostatic charge-discharge profiles of the anode-free full cells using the commercial Cu foil and Cu–10.

The cycling stability of the anode with a limited Li source is evaluated through the symmetric cell, as shown in Figure 6a. The Cu foil electrodes were pre-deposited at 6 mAh cm^−2^ Li before being assembled for symmetric cells. Under a current density of 1 mA cm^−2^ with a capacity of 2 mAh cm^−2^, the polarization of the symmetric cell with the commercial Cu foil electrodes obviously increases after cycling for 400 h. The overpotential even reaches 0.4 V after 600 h, demonstrating the formation of the unstable interphase. However, the symmetric cell with the Cu–10 electrodes can run for 1100 h and always maintain the overpotential below 25 mV. The improved interphase stability derives from the modified Cu–10 current collectors. To further verify the modification effect, the anode-free full cells with 3.5 mAh cm^−2^ LFP cathodes and Cu foil anodes were assembled for the test. All the Li source comes from the cathode and the N/P ratio is 1 in the anode-free battery; thus, any Li loss caused by the unstable interphase will reflect in the capacity drop of the full cell. In the Cu–10||LFP full cell, the Li released from the LFP cathode reacts with the Cu_2_O on the Cu–10 firstly and then forms metal Li during the first charging process. The Cu_2_O transforms into Cu and Li_2_O after Li deposition. It is irreversible during the first discharging process. Thus, the initial coulombic efficiency of Cu–10||LFP cell is lower than that of the Cu||LFP cell. As shown in Figure 6b,c, the anode-free full cell with the commercial Cu foil delivers an initial discharge capacity of 137 mAh g^−1^, and it retains at 59 and 42 mAh g^−1^ with capacity retentions of 43% and 31% after 100 and 200 cycles, implying that the active Li is sharply consumed during the cycling process. As for the anode-free full cell with the Cu–10, it delivers a lower initial discharge capacity of 129 mAh g^−1^, but it maintains 80 and 61 mAh g^−1^ with capacity retentions of 62% and 47% after 100 and 200 cycles. The average values of the coulombic efficiencies of Cu–10||LFP cell are 99.1% and 99.3% during the first 100 and 200 cycles, higher than that of 98.7% and 99.1% of the Cu||LFP cell. The great promotion of the electrochemical performance of anode-free batteries demonstrates that the heat treatment of the Cu foil is beneficial to the establishment of the stable anode interphase although a small amount of active Li will be lost at the initial cycles. In addition, the electrolyte of 1M LiTFSI in DME-DOL + 2%LiNO_3_ we used here is friendly to lithium metal anode for the moderate side reaction and favorable SEI. Thus, the dead lithium formation is reduced to some extent and better electrochemical performance is obtained. However, the electrolyte is still restricted by the inflammability and narrow electrochemical window. For better application, other electrolytes with modified lithium salts or solvents should be tested and less Li loss is expected during the Li plating/stripping in the future works [44,45,46].

## 3. Materials and Methods

### 3.1. Heat Treatment of Cu Foil

The commercial Cu foil in a crucible was transferred into the pre-heated muffle furnace and heated at 320 °C for 5, 10, and 15 min in the atmosphere, respectively (denoted by Cu–5, Cu–10, and Cu–15 in the following). Then, the Cu foil was immediately removed from the muffle furnace so as to avoid excessive oxidation. The heat-treated Cu current collectors were obtained after they were cut into circular pieces.

### 3.2. Characterization

The morphology of Cu foils and Li deposition morphology on the Cu foils were characterized by a field-emission scanning electron microscope (FSEM, Sirion 200 and Quanta 650 FEG, Eindhoven, Holland). The Raman spectra of the heat-treated Cu foils were performed by a confocal Raman microscope (LabRAM HR800, Paris, France) with a 532 nm excitation from an argon-ion laser. The X-ray diffraction (XRD) patterns of the Cu foils were obtained on the equipment (PANalytical B.V., x’pert3 powder) using Cu Kα radiation. An atomic force microscope (AFM, SPM9700, Shimadzu, Japan) was used to measure the roughness of the Cu foils. X-ray photoelectron spectroscopy (XPS, AXIS SUPRA+, Shimadzu-Kratos, Japan) was performed to explore the composition of SEI.

### 3.3. Electrochemical Measurements

CR2032 coin cells were assembled for all electrochemical tests at 25 °C. Celgard 2400 polypropylene membranes were used as separators and 1 M lithium bis(trifluoromethanesulfonyl)imide (LiTFSI) solution in 1,3-dioxolane (DOL) and dimethoxymethane (DME) (1:1 by volume) with 2 wt% LiNO_3_ was used as the electrolyte. The same amount of 50 μL electrolyte was added to each cell. For cathode preparation, the LFP power, conductive carbon and polyvinylidene fluoride (PVDF) binder were mixed at the weight ratio of 93:2.5:4.5 with the solvent of N-methylpyrrolidone. The homogeneous slurry was then cast on carbon-coated Al foils and dried at 70 °C overnight. The mass loading of LFP was about 20–24 mg cm^−2^. For the Li|Cu half-cells in the Coulombic efficiency (CE) tests, 3 cycles cycling between 0.01–0.5 V were performed at 0.1 mA cm^−2^ for activation, then a certain amount of Li was deposited on different Cu foils and stripped up to 0.5 V at different current densities. For the symmetric cells, two Cu foil electrodes with 6 mAh cm^−2^ pre-deposited Li were assembled for tests. The anode-free LFP|Cu full cells were charged at 0.2 C and discharged at 0.5 C between 2.5 and 3.8 V.

## 4. Conclusions

In summary, a high-performance AFLMB with a stable anode interphase is realized through the modification of the Cu foil current collector via a facile one-step heat treatment method. The more stable anode interphase is established on the modified Cu–10 due to the following reasons: (1) The smoother surface morphology after the heat treatment homogenizes the charges and the Li-ions flux. (2) The promoted lithiophilicity may improve the Li nucleation and Li deposition; (3) the generated Cu_2_O on the surface facilitates the Li_2_O-riched SEI, while Li_2_O can regulate Li^+^ transfer and strengthen the structural modulus of SEI. As a result, the cycling stability of the Li|Cu half-cell in the CE test and the symmetric cell is greatly enhanced. Noticeably, the anode-free LFP|Cu full cell with the modified current collector exhibits superior electrochemical performance in the case of the limited Li source, realizing high capacity retentions of 62% and 47% after 100 and 200 cycles. This work provides insights into a potential route toward the current collector design for AFLMB or the other Li metal batteries.

## Data Availability

Not applicable.
